# Prediction of one-day creatinine excretion in Japanese schoolchildren based on anthropometric measurements

**DOI:** 10.1265/ehpm.25-00250

**Published:** 2025-12-04

**Authors:** Sayaka Kato, Yuki Ito, Asako Nakagawa, Kyoko Minato, Mst Sarmin Aktar, Mio Miyake, Shogo Nakane, Michihiro Kamijima

**Affiliations:** Department of Occupational and Environmental Health, Nagoya City University Graduate School of Medical Sciences, 1 Kawasumi, Mizuho-cho, Mizuho-ku, Nagoya 467-8601, Japan

**Keywords:** Creatinine excretion, Predictive equation, Japanese schoolchildren, Urinary excretion, Bland–Altman plot

## Abstract

**Background:**

The creatinine (Cre) concentration in urine is used as an adjustment factor in chemical exposure and nutritional intake assessments. Because Cre excretion varies by sex, age, race, and anthropometric measurements such as height and weight, developing a method suitable for estimating one-day Cre excretion is necessary. Accordingly, this study aimed to develop a predictive equation for individual one-day Cre excretion in Japanese school children.

**Methods:**

Urine samples were collected from 113 boys and 91 girls (aged 6–12 years) from the Aichi Prefecture, Japan, who were free from diseases affecting the renal, muscle, or nervous systems. Urinary concentrations and one-day excreted amounts of Cre were measured and compared with the values obtained using previously reported equations or a fixed value, totaling 11 methods. Subsequently, we developed a new equation using machine learning and multiple regression analyses. Additionally, the estimated one-day sodium excretion value calculated using this equation was compared with the measured value.

**Results:**

Among the 11 methods to predict Cre excretion, 7 overestimated—5 of which showed a positive trend bias with larger differences at higher average concentrations—and 3 underestimated—2 of which showed a negative trend bias with larger differences at lower average concentrations. A new machine learning model using sex, age, and body surface area (calculated from height and weight) yielded the most accurate prediction. Multiple regression analysis, which demonstrated the most accurate prediction, used sex, age, and body surface area as independent variables with or without the first void Cre concentration divided by urination duration from the previous night’s urination to the first void. Moreover, the difference in one-day sodium excretion from first-void urine predicted using our newly developed Cre equation increased as the measured values increased.

**Conclusions:**

Our study suggests that the estimation of one-day Cre excretion based on sex, age, and body surface area is most appropriate for Japanese schoolchildren, particularly in assessing their chemical exposure and dietary nutrient intake.

**Trial registration:**

Trial registration is not applicable as this observational study did not involve any intervention or randomization requiring registration in a clinical trials registry.

**Supplementary information:**

The online version contains supplementary material available at https://doi.org/10.1265/ehpm.25-00250.

## Graphical Abstract

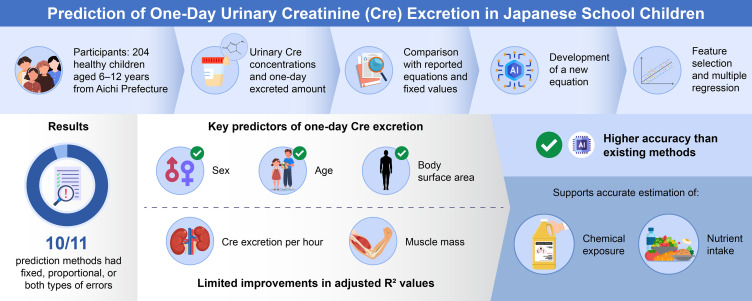

## Background

Daily creatinine (Cre) excretion is constant in each individual; therefore, urinary Cre concentration is typically used to correct for variable dilutions in spot urine samples [1]. By dividing the spot urine concentration of analytes, such as nutrient factors or environmental chemicals, by the spot urine Cre concentration multiplied by the predicted 24-hour Cre excretion, it is possible to predict the 24-hour excretion of analytes [2–6] and assess the total intake of the analytes’ parent compounds.

Cre is a waste byproduct produced in muscles and excreted through urine. Urinary Cre concentration varies considerably with age, sex, ethnicity, and anthropometric factors, including body weight (BW) and height (BH). It is also affected by the amount of muscle or lean mass. Research has revealed that Japanese children in the seventh grade have twice as much lean body mass as those in the first grade [7]. Therefore, the Cre-adjusted analyte concentrations of spot urine samples cannot be directly compared among children of various ages. To date, many studies have presented equations to calculate 24-hour Cre excretion in adults [2, 8–12]; however, similar reports on equations in children are limited [3, 13–15].

In this respect, some epidemiological studies have used age-dependent constant values of 24-hour Cre excretion to assess the intake of nutrients and environmental chemicals from concentrations of analytes in spot urine [16–18]. A 24-hour urine collection is considered the gold standard method as intra-individual within-day variation of chemical excretion in spot urine has been reported [19]. However, this method requires significant effort from children and their parents, particularly because carrying the sample collection cups is inconvenient when going out, although it poses no problem at home. Therefore, the use of the 24-hour Cre excretion estimate equation is a practical and feasible approach to precisely predict 1-day chemical exposure in children and evaluate the effects of nutrient intake and chemical exposure [17, 18]. However, the 24-hour Cre excretion estimate equation for Japanese schoolchildren is limited in scope. In addition, estimation derived from data sets pertaining to children from other countries may not apply to Japanese counterparts owing to variations in muscle mass.

Therefore, we aimed to develop an equation to estimate 24-hour urinary Cre excretion using anthropometric factors in school-aged Japanese children, who are generally more vulnerable to environmental chemicals than adults [20]. The developed equation was used to evaluate the predictability of sodium intake using spot urine samples from a school-aged population in Japan. This equation can contribute to clinical research aiming to prevent illness, such as cardiovascular diseases caused by salt intake in children later in life, through evidence-based policy strategies.

## Methods

### Study population

This study included 113 boys and 91 girls aged 6–12 years. Among them, 131 participated in the Adjunct Study of the Japan Environment and Children’s Study (JECS) and belonged to the Aichi Regional Sub-Cohort of JECS (JECS-A) [21], while the remaining 73 children lived in Aichi but were not JECS participants. Participant recruitment and sample collection were conducted from October 16, 2021, to September 18, 2023.

Study eligibility involved face-to-face screening to ensure they were free from enuresis, nocturnal urine leakage, or any muscle, renal, or neurological disease that could affect Cre excretion. Further, individuals with missing urine samples and those with 1-day-collected urine samples of less than 254 mL were excluded.

All the participants’ guardians provided written informed consent for participation. Furthermore, the study was approved by the Institutional Review Board of Nagoya City University (approval number: 60-21-0126).

### Measures

#### Urine collection and urinalysis

Participants and their guardians were informed about the 24-hour urine collection procedure. The consenting guardians received plastic cups for urine collection and a paper form to record the collection time. The guardians were asked to answer a questionnaire on their children’s medical history.

Participants were required to urinate into the cups each time with the help of their guardians. The first void urine (FVU) on the first survey day was not collected. Starting from the second void urine (SVU), a single complete dose of urine was collected in the cup each time. Unlike on the first day, the second-day FVU was collected.

Subsequently, the participants were required to write down the time of urination on the recording paper. The collected urine was stored at room temperature and kept away from direct sunlight until it was received by the staff. Sample collection involved no restrictions on behavior or medical treatment. The cups containing urine were brought to the survey location or collected by a staff member at the children’s house. Subsequently, these urine samples were stored at 4 °C in a refrigerated room.

Each spot urine volume was measured, and the daily urine excretion volume was calculated within 3 days of FVU collection. Further, each urine sample taken from the same percentage of voided volume was mixed as 24-hour urine. Subsequently, the specific gravity was measured using Atago UG-D (Atago, Osaka, Japan). The concentrations of sodium (Na), potassium (K), and Cre in specific spot urine (FVU, SVU, and third urine collected after 3:00 pm [TVU]) and 24-hour urine were analyzed using H.U. Frontier, Inc. (Tokyo, Japan), a contract laboratory.

#### Anthropometric variables

The BH, BW, and body composition (muscle and fat mass) of all participants were measured. BH was measured twice using SECA 213 (stadiometer; SECA, Hamburg, Germany), and the average of the two values was recorded when the difference between them was less than 1 cm. Contrarily, when the difference was more than 1 cm, BH was measured again, and the average of the two closest values was recorded. BW, muscle mass (expressed in kg), and fat percentage were measured in a single session using Tanita MC780A Body Composition Analyzer (Tanita, Tokyo, Japan). These anthropometric indices were measured by well-trained staff.

### Statistical analysis

#### Selecting variables using machine learning

The study’s complete dataset (n = 194) was created after excluding 10 participants: two children of unknown age and eight children for whom the time of urination was not recorded, making TVU identification impossible. The dataset was divided into 80% and 20% for training and validation, respectively.

We used 26 variables to develop machine learning models, selected based on their potential relevance to muscle mass, possible association with Cre excretion, or feasibility in large cohort studies. These included demographic, anthropometric, and urinary factors described in Supplementary Information, along with direct measures of muscle mass. Body surface area (BSA) was calculated as BSA = √ (BH × BW/3600) [22]. Z scores for BH, BW, and body mass index (BMI) were calculated using the LMS method [23, 24]. Moreover, we considered two additional sets of variables, with and without muscle mass, as features.

To build the prediction model, we first used the training data to select the most relevant features. Different combinations of these selected features were then tested to identify the set that provided the best prediction accuracy. Finally, the performance of the model with the selected features was evaluated using the test data. Model performance was primarily assessed using the coefficient of determination (R^2^). The overview of the feature selection and model evaluation process is presented in Supplementary Fig. 1 with full technical details. The detailed methodology of machine learning is also described in the Supplementary Information.

#### Estimation with multiple regression analysis

Multiple regression analysis was performed to create an estimated equation using the variables selected by machine learning. Further, we selected 10 models from the highly adjusted coefficients of determination and compared the results with those of machine learning.

#### Comparison of measured values with reported estimations and with estimations derived from our results using Bland–Altman plots

The measured 24-hour Cre excretion values were compared with the estimated values derived from 10 reported equations for Cre excretion estimation [5, 13–15, 18, 21, 25, 26]. Another estimation method for comparison was the use of a fixed value (800 mg) as one-day Cre excretion. We selected the value based on the median concentration of Cre in our participants’ FVU, a one-day urine volume of 800 mL (the typical daily urine volume in schoolchildren aged 8–14 years; range: 800–1400 mL [27]), and the assumption that Cre excretion would have been constant over the 24 hours of sample collection. Further, Bland–Altman analyses were conducted to evaluate the validity of each estimation method by assessing fixed errors and proportional trend errors. A fixed error indicates that a given method constantly under- or over-estimates compared to the other one. A proportional error indicates that the same differences are constant, correlating with solute concentration levels [28].

We also used Bland–Altman plots to compare measured values with those estimated by the original multiple regression equation in order to evaluate and refine the model, and further compared measured values with those estimated by the improved prediction equation.

#### Verification of sodium excretion prediction

As an example of applying one-day Cre excretion, we performed the sodium excretion prediction using our regression machine learning results and the equation presented by Tanaka et al. [16], which was developed to estimate sodium excretion in school-aged children from spot urine samples. The prediction was based on three different spot urine samples: FVU, SVU, and TVU. The relevant equation is as follows:

$$\begin{array}{rl} & \text{Predicted sodium excretion}\;(\text{mEq/day})\\ & \;=21.98\\ & \;\times (\text{spot urine sodium concentration}\;(\text{mEq/L})\\ & \;/\text{spot urine creatinine concentration}\;(\text{mg/dL})/10\\ & \;\times \text{one-day creatinine excretion}\;(\text{mg/day}){)}^{0.392} \end{array}$$

Further, we used Bland–Altman plots to compare each predicted value with the measured sodium excretion value.

#### Software for statistical analysis

For all statistical analyses except machine learning, we used R (version 4.3.1; R Foundation for Statistical Computing, Vienna, Austria). Bland–Altman plots were made using the “blandr” package (version 0.6.0. Deepankar Datta, UK) [28]. Finally, Python (version 3.9.13; Python Software Foundation, Wilmington, DE, USA), “scikit-learn” (library version 1.2.1; scikit-learn developers, INRIA, Paris, France), and “mlxtend” library (version 0.23.0; Sebastian Raschka, Madison, WI, USA) were used for machine learning.

## Results

### Participant characteristics

Table 1 lists the participants’ characteristics. Their average age was 8.7 (standard deviation (SD), 1.5) years (109.6 (SD 17.3) months). The average muscle percentage of boys was significantly higher than that of girls, although there were no significant sex-related differences (p > 0.05) in BH, BW, BMI, and BSA. Additionally, no significant sex-related differences were observed in urine concentrations of Cre, sodium, potassium, as well as urine specific gravity or volume. The median Cre concentration in FVU among the participants was 104 mg/dL.

**Table 1:** Participant characteristics (n = 204)

	**All** **(n = 204)**	**Boys** **(n = 113)**	**Girls** **(n = 91)**	**p-value**
Age (year)	8.7 ± 1.5	8.7 ± 1.4	8.6 ± 1.6	0.56
Age (month)	109.6 ± 17.3	109.8 ± 16.2	109.4 ± 18.6	0.88
Height (cm)	131.3 ± 10.9	132.5 ± 9.9	129.8 ± 11.8	0.07
Weight (kg)	28.6 ± 7.2	29.3 ± 7.2	27.6 ± 7.0	0.08
Body mass index (kg/m^2^)	16.3 ± 2.3	16.5 ± 2.6	16.1 ± 1.9	0.18
Body surface area (BSA) (m^2^)	1.02 ± 0.16	1.04 ± 0.15	0.99 ± 0.17	0.07
Fat (%)	14.6 ± 6.7	13.8 ± 7.9	15.7 ± 4.7	**<0.05**
Muscle (kg)	22.9 ± 4.5	23.7 ± 4.2	21.9 ± 4.7	**<0.01**
CreU_24_ (mg/dL)	71.87 [55.44–92.24]	73.58 [56.56–94.19]	69.45 [53.30–88.45]	0.18
NaU_24_ (mEq/L)	148.5 [110.8–189.0]	159 [113.5–194.0]	138 [105.5–175.0]	0.06
KU_24_ (mEq/L)	42.5 [32.20–55.38]	45.4 [33.00–58.40]	39.7 [30.50–51.50]	0.07
SG_24_	1.02 [1.015–1.025]	1.021 [1.016–1.026]	1.019 [1.015–1.024]	0.1
UV_24_ (mL)	686 [531.6–922.5]	697 [555.0–967.0]	648 [512.5–888.5]	0.26

SG_24_: specific gravity of 24-hour urine; UV_24_: urine volume

Values are reported as mean ± standard deviation or median [interquartile range]. p-values are based on Mann–Whitney U test. CreU_24_, NaU_24_, and KU_24_ values indicate the concentrations of creatinine, sodium, and potassium in 24-hour urine, respectively.

BSA = √ ((Height * Weight)/3600)

Bold font indicates p-values < 0.05.

### Comparison of the Cre estimates obtained using reported equations with actual values

**Fig. 1:**
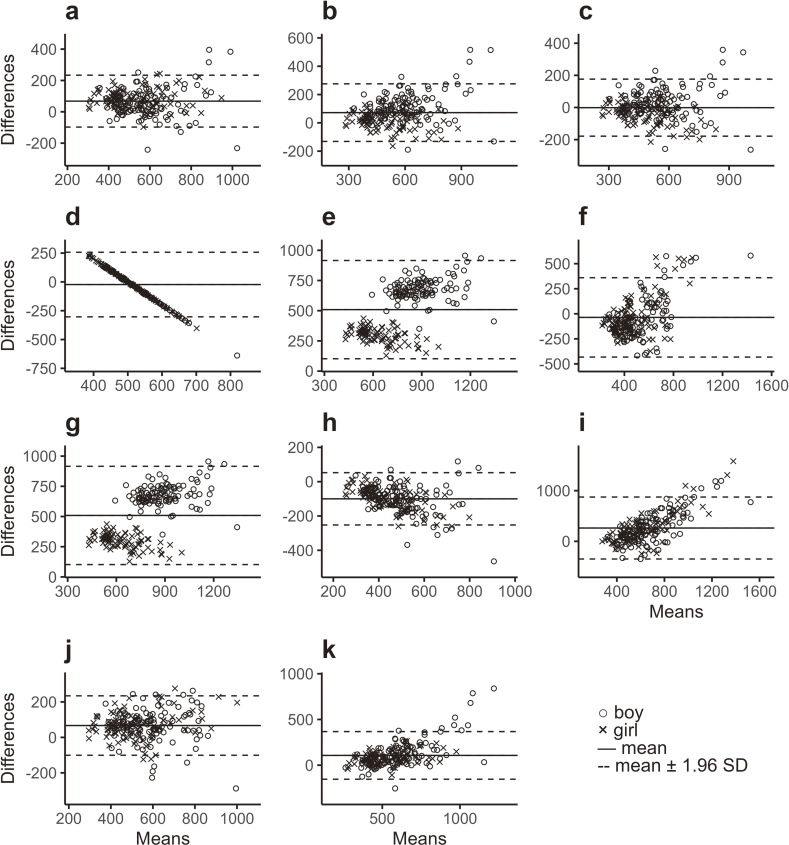
Bland–Altman plots between estimated and measured 24-hour creatinine excretion. Estimated creatinine excretion was derived from the calculation presented in Table 2. The horizontal axes indicate the mean values of estimated and measured excretion, whereas vertical axes depict differences between estimated and measured excretion. Symbols and lines are explained within the figure.

In this study, Bland–Altman analyses, which indicate mean differences, trend bias, and a 95% limit of agreement (LOA), revealed biases indicating fixed errors, proportional errors, or both in 10 of 11 Cre predictive methods (Methods A–K) (Table 2, Fig. 1). A fixed error was not observed in Method C, in which the calculation was based on BW alone. Further, a proportional error (a significant trend bias) was not observed in Methods A and C, both of which involved calculations using BW alone. In some other methods (B, D–I, and K), both error types were observed.

**Table 2:** Brand–Altman analysis results between actual creatinine excretion levels and estimates from reported equations

**Method**	**Sex**	**Calculation for creatinine excretion (mg/day)**	**Mean difference**	**Trend bias**	**95% LOA**	**Reference citation**
			**(95% CI)**	**(p)**		
A	Both	BW × 20.7	68	0.04	−97.5 to 234.0	[14]
			(56.4, 79.7)	−0.37		
B	Boys	BW × 23	72.2	0.21	−131.4 to 275.8	[22]
	Girls	BW × 18	(57.9, 86.5)	(<0.001)		
C	Boys	BW × 20	−1.1	0.05	−178.6 to 176.3	[14]
	Girls	BW × 16	(−13.6, 11.4)	−0.3		
D	Both	Set estimated creatinine at 800 mg/day	−23.0	−2.0	−303.1 to 257.2	See text
			(−42.7, −3.2)	(<0.001)		
E	Boys	age × (−12.63) + BW × 15.12 + BH × 7.39 − 79.9	508.9	0.72	102.2 to 915.6	[23]
	Girls	age × (−4.72) + BW × 8.58 + BH × 5.09 − 74.95	(480.3, 537.6)	(<0.001)		
F	Both	CreF × 0.015/(SGF − 1)	−36.0	0.65	−431.9 to 360.0	[14]
			(−63.8, −8.1)	(<0.001)		
G	Both	CreF × 0.022/(SGF − 1)	191.3	1	−398.7 to 781.3	[14]
			(149.8, 232.9)	(<0.001)		
H	Both	Estimate using Chinese report	−100.0	−0.28	−252.4 to 52.4	[13]
			(−111.7, −89.3)	(<0.001)		
I	Boys	UV24 × 104.4	262.6	1.1	−348.7 to 874.0	[18]
	Girls	UV24 × 99.48	(219.6, 305.7)	(<0.001)		
J	Boys	BH × 1.085 × [(BH − 168) × 0.0564 + 6.265]	66.8	0.04	−100.8 to 234.4	[5]
	Girls	BH × 1.085 × 2.045 × exp[(BH − 90) × 0.01552]	(55.0, 78.6)	−0.37		
K	Boys	−221 − 0.94 × BH + 35	106.7	0.47	−153.9 to 367.3	[15]
	Girls	−330 + 2.74 × BH + 19.57 × BW	(88.4, 125.1)	(<0.001)		

CI: confidence interval; LOA: limit of area; BW: body weight; BH: body height; CreF: creatinine concentration in first void urine; UV_24_: urine volume; SGF: specific gravity of first void urine.

### Investigation of equations to predict 24-hour Cre excretion

#### Machine learning

Table 3 reports the feature importance ranking results from the dataset without muscle mass as a feature. To construct the Lasso model using training data, we examined how the number of selected features influenced the model’s fit. The best fit, with the highest mean accuracy of 0.727, was achieved when selecting four features from the 26 features under consideration.

**Table 3:** Feature ranking selected by machine learning

**Feature**	**Ranking**
Sex	1
Age (year)	1
BH (cm)	1
BSA (m^2^)	1
CreF (mg/dL)/hour	2
CreT/hour/SG	3
CreF/hour/SG	4
CreS/SG	5
CreT/SG	6
CreF/SG	7
CreT/hour	8
CreF (mg/dL)	9
KF (mEq/L)	10
NaF (mEq/L)	11
BMI (kg/m^2^)	12
BW (kg)	13
BH Z score	14
Interval to first void urine (hour)	15
BW Z score	16
SGF	17
BMI Z score	18
CreS (mg/dL)	19
SGT	20
SGS	21
CreT (mg/dL)	22

BH: body height; BSA: body surface area; BW: body weight; CreF: creatinine concentration of first void urine; CreS: creatinine concentration of second void urine; CreT: creatinine concentration of urine voided after 3 PM; SGF: specific gravity of first void urine; SGS: specific gravity of second void urine; SGT: specific gravity of urine voided after 3 PM; hour: excretion interval from last excretion; KF: Potassium concentration of first void urine; NaF: Sodium concentration of first void urine; BMI: body mass index.

We selected sex, age, BH, BSA, and hourly Cre excretion rate in FVU (CreF/h), each with an importance ranking of 1 or 2. We then compared the models using machine learning to ensure that at least one of the selected features was retained. The model included sex, age, and BSA, which was selected because it had the highest adjusted coefficient of determination (Table 4), indicating parameter pre-tuning results. After performing 20-fold cross-validation and Lambda parameter tuning, the coefficient of determination of the model with sex, age, and BSA was 0.717 in the training data. Further, when applied to the test data, the coefficient of determination was 0.655 with cross-validation. Additionally, we investigated the model including muscle as a feature, and the cross-validated coefficients of determination for the training and test data were 0.724 and 0.709, respectively.

**Table 4:** Feature names and scores and adjusted scores for the training data

**Model no.**	**Feature name**	**Average score of the coefficient ** **of determination**	**Average score of the adjusted coefficient ** **of determination**
1	Sex, age, BSA	0.731	0.725
2	Sex, age, BSA, CreF/hour	0.731	0.724
3	Sex, BSA	0.724	0.721
4	Sex, BSA, CreF/hour	0.725	0.720
5	Sex, age, BH, BSA	0.727	0.720
6	Sex, age, BH, BSA, CreF/hour	0.727	0.718
7	Sex, BH, BSA	0.722	0.717
8	Sex, BH, BSA, CreF/hour	0.724	0.717
9	Age, BSA	0.717	0.713
10	Age, BSA, CreF/hour	0.717	0.712

BH: body height; BSA: body surface area; CreF: creatinine concentration of first void urine; CreF/hour: creatinine concentration of first void urine.

#### Regression analysis

The regression analysis model included sex, age, BH, BSA, and CreF/h as the result of machine learning. Table 5 shows the intercepts, coefficient, and adjusted R-squared value for each model. The adjusted R-squared of Model 1, which included sex, age, and BSA, was 0.764. Model 2 included CreF/h and the variables of Model 1 and had an adjusted R-squared of 0.769. The intercepts and coefficients of all models were statistically significant.

**Table 5:** Regression coefficients and adjusted R^2^

**Model no.**	**Intercept** **(p)**	**Sex** **boy;1** **girl;2**	**Age** **(year)**	**BH**	**BSA**	**CreF/h**	**Adjusted R^2^**
1	**−198.96**	**−38.54**	**15.43**		**633.73**		0.764
	(<0.001)	(<0.001)	(<0.01)		(<0.001)		
2	**−220.22**	**−37.96**	**13.77**		**642.37**	**2.39**	0.769
	(<0.001)	(<0.001)	(<0.01)		(<0.001)	(<0.05)	
3	**−186.30**	**−35.72**			**748.55**		0.755
	(<0.001)	(<0.001)			(<0.001)		
4	**−211.97**	**−35.39**			**744.37**	**2.7**	0.763
	(<0.001)	(<0.001)			(<0.001)	(<0.01)	
5	**−291.47**	**−37.61**	11.84	1.37	**576.72**		0.764
	(<0.001)	(<0.001)	−0.06	−0.28	(<0.001)		
6	**−342.59**	**−36.70**	9.01	1.79	**568.59**	**2.6**	0.771
	(<0.001)	(<0.001)	−0.14	−0.15	(<0.001)	(<0.01)	
7	**−369.21**	**−35.18**		**2.63**	**588.61**		0.761
	(<0.001)	(<0.001)		(<0.001)	(<0.001)		
8	**−404.67**	**−34.82**		**2.75**	**576.55**	**2.8**	0.769
	(<0.001)	(<0.001)		(<0.01)	(<0.001)	(<0.01)	
9	**−267.86**		**13.42**		**663.72**		0.747
	(<0.001)		(<0.05)		(<0.001)		
10	**−288.84**		**11.74**		**672.22**	**2.5**	0.753
	(<0.001)		(<0.05)		(<0.001)	(<0.05)	

BH: body height (cm); BSA: body surface area (m^2^); CreF: creatinine concentration in first void urine (mg/dL).

p-values are provided in parentheses. Values in bold indicate statistical significance at p < 0.05.

**Fig. 2:**
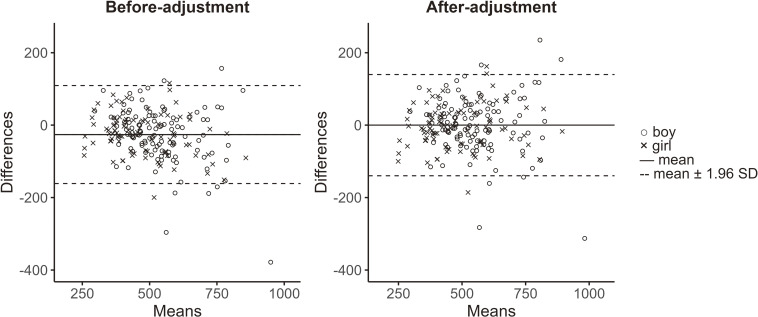
Bland–Altman plots between estimated and measured 1-day creatinine excretion. Estimated creatinine excretion was obtained using the equation Model 1R. The horizontal axes indicate the mean values of estimated and measured excretion. The vertical axes indicate the differences between estimated and measured excretion. Symbols and lines are explained within the figure.

When Model 1 regression was used as an equation, the mean of differences, 95% LOA, and trend in the Bland–Altman plot were −26.0 mg/dL, −161.2 to 109.3, and 0.15, respectively (Fig. 2, left panel). Then, the trend bias was adjusted as follows:

$$\begin{array}{rl} & \text{Adjusted equation to estimate Cre excretion}\;(\text{mg/day}):\\ & \;[\text{Equation Model 1R}]\\ & \;=[\text{Equation Model 1}]*(1+0.15)-49\\ & \;=-277.40-\text{sex}*44.32+\text{age}*17.74+\mathrm{BSA}\\ & \;\;*728.79 \end{array}$$

where

- sex is coded as 1 for boys and 0 for girls
- age is in years
- BSA (body surface area) is calculated using Mosteller’s formula [26]:
$$\begin{array}{r} \mathrm{BSA}=\sqrt{\mathrm{BH}*\mathrm{BW}/3600} \end{array}$$
- BH is body height in centimeters
- BW is body weight in kilograms
- Cre excretion is expressed in milligrams per day (mg/day)

Figure 2 (right panel) depicts the Bland–Altman plot following the adjustment of the equation. The mean of differences, 95% LOA, and trend in the plot were −0.015 mg/dL, −139.7 to 139.7, and 0.00089, respectively.

#### Sodium excretion

**Fig. 3:**
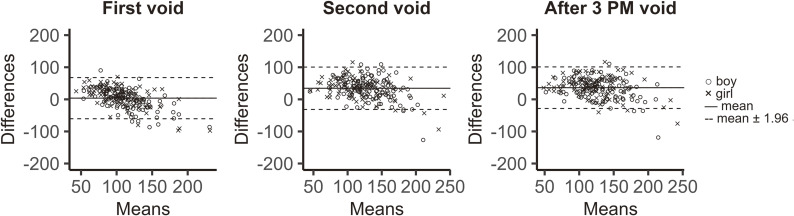
Bland–Altman plots between estimated and measured 24-hour sodium excretion. Sodium excretion was estimated using the method reported by Tanaka et al. [16]. One-day creatinine excretion calculated using our regression analysis results, based on participants’ sex, body height, and body weight. Symbols and lines are explained within the figure.

The median of measured sodium excretion was 148.5 mEq/day. After obtaining predicted sodium excretion using FVU, SVU, or TVU using Tanaka’s equation [16], Bland–Altman analyses were conducted (Fig. 3). The mean differences (fixed error) in the Bland–Altman plots for FVU, SVU, and TVU were 3.7, 34.5, and 36.3 mEq/day, respectively. However, proportional errors were observed for all three types of spot urine samples as well (Fig. 3).

## Discussion

In this study, we formulated a predictive equation (Model 1R) using sex, age, and BSA as variables to estimate one-day Cre excretion based on feature selection and model development from machine learning. Although 26 variables were initially considered, we employed Lasso regression—a machine learning-based method—specifically suited to high-dimensional data. Unlike classical regression, which requires the sample size to be much larger than the number of predictors, penalized regression methods such as the Lasso operate under a much weaker condition. According to Wainwright [29], the required sample size for reliable variable selection in our setting would be about 25 or more. In contrast, our dataset contained 209 subjects, exceeding this threshold by a wide margin. This provides strong support that our modeling strategy is statistically well-founded despite the modest overall sample size. This approach is consistent with the well-known finding that Cre excretion is influenced by age, race, BH, BW, muscle mass, and sex [1–3, 5, 30–34]. Predictions using BW or BSA, which are calculated from BW and BH, are particularly suitable for Japanese children. The finding by Remer et al. [3] that children’s daily Cre excretion rates are better predicted using BSA and age than using BW alone is consistent with our results. Interestingly, our regression analysis indicated that the incorporation of CreF/h into models (Models 2, 4, 6, 8, and 10) did not significantly improve adjusted R-square values. Similarly, the models incorporating muscle mass indicated only slight improvements in adjusted R-square values (data not shown). Further, Cre excretion is strongly linked to muscle metabolism, and CreF/h reflects urinary excretion dynamics over time. Therefore, the current findings were unexpected because we anticipated that the incorporation of both muscle mass and CreF/h would theoretically lead to a substantial improvement in predictive accuracy. These could possibly be explained by the lower variability in lean mass among younger children compared with older children [35].

Though models incorporating muscle mass or CreF/h indicated slight improvements, muscle mass measurement involves bioelectrical impedance analysis, and CreF/h calculation requires the time recording of urine collections, potentially increasing the burden of data collection and likelihood of missing data. Therefore, predictive equations for one-day Cre excretion with CreF/h or muscle mass are largely impractical in epidemiological studies [36, 37]. Accordingly, we recommend the use of the equation Model 1R to balance accuracy and feasibility for Japanese schoolchildren. Because Cre excretion is lower in children and older adults compared to other age groups; in Asians than in Europeans; in individuals with amputations, paraplegia, and muscular dystrophy compared to healthy individuals; and in women compared to men [38], adjusting by sex, anthropometric data, and participants’ characteristics (including country) is important when estimating Cre excretion. This study provides the first Cre excretion equation for school-aged children developed using a machine-learning approach with cross-validation, which enabled the selection of the optimal combination of variables and improved prediction accuracy for Japanese schoolchildren. While the equation improved prediction accuracy for Japanese schoolchildren, its applicability to children of other nationalities—whose body composition may differ—remains to be confirmed. Further validation studies in diverse populations are warranted to assess the study’s generalizability.

We applied 11 methods of 1-day Cre excretion prediction in Japanese children, including 10 previously reported methods [5, 13–15, 18, 25, 26] and one additional method—the use of a selected fixed value (800 mg/day). Among the 11 methods, Method C had the smallest mean difference and exhibited no systematic bias, making it the most suitable. We compared the predicted values obtained using this method and our participants’ 24-hour Cre excretion values. The 95% LOA of the Bland–Altman plot comparing our method with the variables sex, age, and BSA (Equation Model 1R) was smaller than that of Method C. Therefore, our estimation equation potentially reduces the misclassification of chemical exposure and nutrient intake levels. Further, plot results revealed that Method J, originally developed by Mage et al. [5], had a fixed bias in our study participants (elementary school students), although Okuda et al. reported it to be a good fit for junior high school students [39]. Children’s age can affect their Cre excretion; therefore, this result indicates the importance of estimating the children’s Cre excretion using methods different from those used for adults. Additionally, some researchers developed Method K [40] for Japanese children aged 1–18 years; however, it had fixed and trend biases. This is probably due to differences in fat percentage because their 8-year-old participants had a lower fat percentage than our participants. Additionally, this study was conducted approximately 40 years ago, and the average body composition of Japanese children has changed over the years [41]. Along with fat percentage differences, height and weight variations might have reflected muscle mass changes, contributing to the observed bias.

Sodium excretion testing is important for the prevention of hypertension, cardiovascular disease, and cerebrovascular disease later in life. Therefore, we calculated one-day sodium excretion with our Cre prediction method and Tanaka’s method [16] using FVU, SVU, and TVU values. Our results revealed that with the increase in one-day measured sodium excretion, the sodium excretion estimated from a spot urine sample tended to decrease below the actual measured value, indicating a systematic trend bias. Further, researchers reported on the estimates of children’s salt intake using Tanaka’s method [42–46]. However, these reports did not clarify the differences in Cre excretion between adults and children or any differences based on sex. Similarly, the use of equations assessed in pediatrics was not found satisfactory for assessment at the individual level by Dong et al. [47], although Tanaka’s equation offered a plausible alternative to the average population’s 24-hour sodium estimation for young adolescents. Moreover, our results suggest that Tanaka’s method is not appropriate for sodium excretion estimation in Japanese children and the predicted intake may underestimate actual values in case of high sodium intakes.

This study has some limitations. First, only 34 (17%) participants were older than 10 years. Because fat and muscle mass proportions change in growing children, including during puberty, the generalizability to older schoolchildren may be limited, and more individuals aged 10 years or older should be studied to validate our equation for such children. Second, the time of FVU on the first day was not recorded; therefore, the duration of urine collection could not be adjusted. Finally, food intake was not determined in this study, although meat consumption is known to affect Cre excretion [48], and future studies should incorporate diet records (e.g., 24-hour recalls or food frequency questionnaires) or dietary biomarkers to better account for this influence. Despite these limitations, our findings facilitate the accurate estimation of nutritional intake and chemical exposure and thereby prevent any misclassification in these assessments in Japanese children.

## Conclusions

Our study suggests that the estimation of 24-hour Cre excretion based on sex, age, and BSA calculated from BH and BW is most suitable for Japanese elementary school children. Compared to existing methods, our equation better accounts for age-specific variations in Japanese elementary school children, enhancing exposure assessment and nutritional research.

## Abbreviations

BH: body height
BMI: body mass index
BSA: body surface area
BW: body weight
Cre: creatinine
CreF: creatinine concentration in first void urine
CreF/h: creatinine concentration in first void urine divided by the excretion interval of first void urine
FVU: first void urine
JECS: the Japan Environment and Children’s Study
JECS-A: Aichi Regional Sub-Cohort of the Japan Environment and Children’s Study
SVU: second void urine
TVU: third urine collected after 3:00 pm

## Supplementary information

**Additional file 1:** **Supplementary Figure 1.** Machine learning method.

**Additional file 2:** Machine learning method.

## Declarations

### Availability of data and materials

Data cannot be shared for privacy or ethical reasons.
