# Supplementary figures and images for: Prediction of one-day creatinine excretion in Japanese schoolchildren based on anthropometric measurements
Source: Environ Health Prev Med. 2025 Dec 4;30:97. doi: 10.1265/ehpm.25-00250 (PMC12698365; doi:10.1265/ehpm.25-00250)

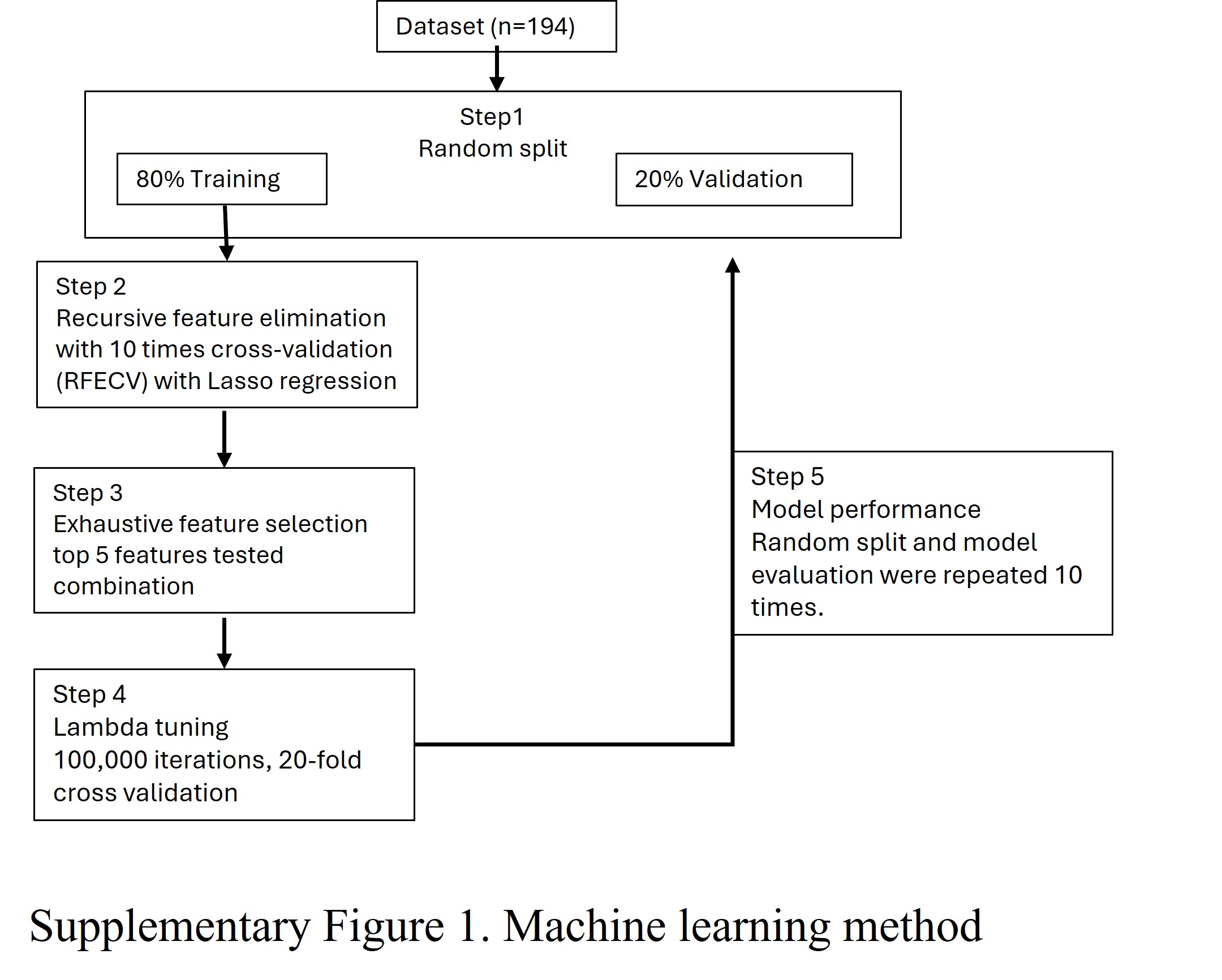

Supplement: Supplementary file 1 — Additional file 1: Supplementary Figure 1. Machine learning method. [file ehpm-30-097-s001.jpg]
