# Supplementary material for: Prediction of one-day creatinine excretion in Japanese schoolchildren based on anthropometric measurements
Source: Environ Health Prev Med. 2025 Dec 4;30:97. doi: 10.1265/ehpm.25-00250 (PMC12698365; doi:10.1265/ehpm.25-00250)
Supplement: Supplementary file 2 — Additional file 2: Machine learning method. [file ehpm-30-097-s002.docx]

**Machine learning method**

The study’s complete dataset (n=194) was created after excluding 10 participants: two children of unknown age, and eight children for whom the time of urination was not recorded, making the identification of the urine collected after 3:00 pm (TVU) impossible. The dataset was divided into 80% and 20% for training and validation, respectively.

We used 26 variables to develop machine learning models. These included: sex; age (year); body height (cm); body weight (kg); body height z-score; body weight z-score; body mass index (kg/m^2^); body mass index z-score; body surface area (m^2^); creatinine concentration of first void urine (FVU, mg/dL); creatinine concentration of second void urine (SVU, mg/dL); creatinine concentration of TVU (mg/dL); creatinine concentration of FVU divided by the excretion interval; creatinine concentration of TVU divided by the excretion interval; creatinine concentration of FVU divided by both the excretion interval and specific gravity; creatinine concentration of TVU by both the excretion interval and specific gravity; creatinine concentration of FVU divided by specific gravity; creatinine concentration of SVU divided by specific gravity; creatinine concentration of TVU by specific gravity; specific gravity of FVU; specific gravity of SVU; specific gravity of TVU; interval to first void urine (hour); potassium concentration of FVU (mEq/L); and sodium concentration of FVU (mEq/L). Moreover, we considered two additional sets of variables, with and without muscle mass, as features.

After normalizing the feature values, we performed recursive feature elimination with cross-validation (RFECV)—a feature selection technique that uses a recursive process to rank features according to their importance and applies elimination to exclude weak features, dependencies, and collinearities from a model to improve its prediction accuracy. We performed RFECV and the selection operator (Lasso) model and tested the features using 10-fold cross-validation. These features were evaluated based on their coefficients of determination. Subsequently, we performed exhaustive feature selection (EFS) with at least one among the top five features. Finally, we performed 20-fold cross-validation with 100,000 iterations for λparameter tuning during model training to select the optimal value for the Lasso method. Following feature selection, a 10-fold cross-validation technique was used to assess the model using the test data.
